# Utilisation of complementary and alternative medicine (CAM) practitioners within maternity care provision: results from a nationally representative cohort study of 1,835 pregnant women

**DOI:** 10.1186/1471-2393-12-146

**Published:** 2012-12-12

**Authors:** Amie Steel, Jon Adams, David Sibbritt, Alex Broom, Cindy Gallois, Jane Frawley

**Affiliations:** 1 Faculty of Health, UTS, Level 7, Building 10, 235-253 Jones Street, Ultimo, New South Wales, 2006, Australia; 2 School of Social Science, University of Queensland, St Lucia, Queensland, 4072, Australia

**Keywords:** Pregnancy, Complementary medicine, Health services, Interprofessional, Integrative medicine

## Abstract

**Background:**

There is little known about women’s concurrent use of conventional and complementary health care during pregnancy, particularly consultation patterns with complementary and alternative medicine (CAM). This study examines health service utilisation among pregnant women including consultations with obstetricians, midwives, general practitioners (GPs) and CAM practitioners.

**Methods:**

A sub-study of pregnant women (n=2445) was undertaken from the nationally-representative Australian Longitudinal Study on Women’s Health (ALSWH). Women’s consultations with conventional practitioners (obstetricians, GPs and midwives) and CAM practitioners for pregnancy-related health conditions were analysed. The analysis included Pearson chi-square tests to compare categorical variables.

**Results:**

The survey was completed by 1835 women (response rate = 79.2%). A substantial number (49.4%) of respondents consulted with a CAM practitioner for pregnancy-related health conditions. Many participants consulted only with a CAM practitioner for assistance with certain conditions such as neck pain (74.6%) and sciatica (40.4%). Meanwhile, women consulted both CAM practitioners and conventional maternity health professionals (obstetricians, midwives and GPs) for back pain (61.8%) and gestational diabetes (22.2%). Women visiting a general practitioner (GP) 3–4 times for pregnancy care were more likely to consult with acupuncturists compared with those consulting a GP less often (p=<0.001, *x*^*2*^=20.5). Women who had more frequent visits to a midwife were more likely to have consulted with an acupuncturist (p=<0.001, *x*^*2*^=18.9) or a doula (p=<0.001, *x*^*2*^=23.2) than those visiting midwives less frequently for their pregnancy care.

**Conclusions:**

The results emphasise the necessity for a considered and collaborative approach to interactions between pregnant women, conventional maternity health providers and CAM practitioners to accommodate appropriate information transferral and co-ordinated maternity care. The absence of sufficient clinical evidence regarding many commonly used CAM practices during pregnancy also requires urgent attention.

## Background

### Women’s health and the rise of complementary and alternative medicine

Complementary and alternative medicine (CAM) - a range of treatments and practices not traditionally associated with the conventional medical profession or medical curriculum [1] - is more commonly used by women than men [2,3]. CAM appears to be making its presence felt with regards to a number of women’s health issues [4-7] and, consistent with the wider population [8,9], female CAM users employ these treatments supplementary to conventional care [4].

### Pregnancy and CAM: evidence, communication and risk

Pregnant and birthing women have been identified as substantial CAM users with prevalence rates of between 20% to 60% [5] - a usage suggested to be in line with the search for a ‘natural’ pregnancy and birthing experience (free, where possible, from medical intervention) [10,11]. While a small but emerging body of literature highlights acupuncture/acupressure, aromatherapy, chiropractic, homoeopathy, massage and yoga as popular amongst pregnant women [5,12] opinions differ on the validity and safety of these CAM practices for pregnancy [10,13,14]. Approximately 30% of pregnant women who consult CAM therapists do so without informing their midwife or doctor [15] yet there is also evidence of CAM referral practices in maternity care [16]. CAM referrals during pregnancy are more likely to be midwife-led than obstetrician-led and obstetricians appear more cautious and sceptical than midwives about CAM use for women in their care [16].

Evidence of the efficacy of specific CAM modalities for different pregnancy-related complaints, while emerging, remains scant [17-22]. A systematic review has identified a trend towards improved outcomes for women receiving chiropractic care for pregnancy-related back pain [18]. Acupuncture may be an effective approach for the management of nausea and vomiting [20] and pelvic or back pain in pregnancy [22] and naturopathic recommendations for the treatment of nausea in pregnancy (including ginger and vitamin B6) [14] appear to have some low-level evidence of benefit [21].

### Identifying gaps in CAM use in maternity care research

From within the small yet growing body of research focused upon CAM use for pregnancy [2,5], the majority has examined pregnant women’s use of discrete supplements or treatments and, despite recommendations [23,24], there has been little exploration of women’s consultations with CAM practitioners. One exception is a recent longitudinal cohort study (n=535) [7] which identified no significant change in this prevalence rate over a 10 year period compared with non-pregnant women. Unfortunately, this recent work does not discern the prevalence of pregnant women’s use of specific types of CAM practitioners or examine the patterns of pregnant women’s consultations with CAM practitioners for the purpose of managing pregnancy-related health concerns. Neither does this previous work examine how such consultation patterns relate to the use of conventional maternity care providers, explore the health reasons for which pregnant women consult CAM practitioners or draw from a nationally representative sample of pregnant women. In response to these important knowledge gaps, this study - presenting findings from the largest nationally representative cohort of pregnant women on CAM use to date (n=1835) – aims to provide the first detailed examination of conventional and CAM practitioner use during pregnancy.

## Methods

This research was conducted as part of a sub-study of the Australian Longitudinal Study on Women’s Health (ALSWH) investigating women’s use of health care during pregnancy and birthing, conducted in 2010. The ALSWH was established in 1996, when women in three age groups (‘younger’ 18–23, ‘mid age’ 45–50 and ‘older’ 70–75 years) were randomly selected from the national Medicare database. The ALSWH was designed to examine demographic, social, physical, psychological, and behavioural variables and their effect on major aspects of women’s health and wellbeing. Women from the ALSWH younger cohort, who were aged 31–36 years in 2009 (n=8012) and who identified as being pregnant or as having recently given birth in the 2009 ALSWH survey (n=2445) were identified for inclusion in the sub-study and were surveyed in 2010. Ethics approval for the sub-study was gained from the University of Newcastle ethics committee (#H-2010_0031).

### Demographic characteristics

The women were asked about their marital status, educational qualifications and health insurance cover.

### Health service utilisation

Women were asked about their visits to health care practitioners including conventional maternity care providers (general practitioners (GPs), obstetricians and midwives) and CAM practitioners (acupuncturists, aromatherapists, chiropractors, naturopaths/herbalists, doulas, massage therapists, meditation/yoga practitioners, osteopaths).

### Reasons for use of CAM

Women were asked who they consulted for management of pregnancy-related conditions. Women were also asked to rate their level of satisfaction with a variety of care options for their pregnancy and birth including GPs, obstetricians and midwives.

### Statistical analyses

Pearson’s chi-square tests were used to compare categorical variables. To correct for multiple statistical testing, a modified Bonferroni correction was used [25]. All analyses were conducted using the statistical software Stata 11.2.

## Results

There were 1835 women who completed and returned the questionnaire (RR=79.2%), the majority of which were in a relationship (96.3%) and had tertiary level education (60.1%). The majority of women had current private health insurance (72%), with 58.4% including cover for pregnancy-related care.

### Conventional and complementary health service utilisation during pregnancy

During pregnancy and birth, the women consulted with a diverse range of both conventional maternity care practitioners and CAM practitioners (see Table 1). Almost all women (99.8%) had consulted with a conventional practitioner at some stage during their pregnancy with the most common being a GP (90.6%). Meanwhile, half (49.4%) had consulted with a CAM practitioner of some kind, most commonly with a massage therapist (34.1%), chiropractor (16.3%) and a meditation/yoga practitioner (13.6%).

**Table 1:** **Women**’**s consultations with complementary and alternative medicine** (**CAM**) **and conventional medicine practitioners for pregnancy**-**related health conditions**

**Professional group**	**None**	**1 or 2**	**3 or 4**	**5 or 6**	**7 or more**	**Total**	**Respondents**
	**%**	**%**	**%**	**%**	**%**	**%**	***n***
GP (n = 1734)	9.9	51	14.8	9.5	14.8	90.6	1734
Obstetrician (n=1662)	14.8	13.8	7.2	13.2	51	86.6	1662
Midwife (n=1520)	35.3	20.1	12.5	12.4	19.7	70.7	1520
Any conventional practitioner						99.8	
Acupuncturist (n=1714)	90.6	4	2.4	1.3	1.7	9.5	1714
Aromatherapist (n=1670)	99.4	0.5	0	0.06	0.06	0.6	1670
Chiropractor (n=1709)	83.7	4	4.3	2.8	5.3	16.3	1709
Naturopath/Herbalist (n=1684)	92.8	4.3	1.6	0.8	0.5	7.2	1684
Doula (n=1667)	98.6	0.7	0.4	0.1	0.2	1.4	1667
Massage (n=1743)	65.9	20.6	7.3	3.2	3	34.1	1743
Meditation/Yoga (n=1690)	86.4	2.5	1.7	1.6	7.8	13.6	1690
Osteopath (n=1690)	93.9	2.5	1.2	0.9	1.5	6.2	1690
Any CAM practitioner						49.4	

The women engaged with a number of practitioners concurrently (see Table 2), with a substantial number of participants consulting with two (48.2%) or three (42.2%) types of conventional maternity carers during their pregnancy. In contrast, the majority of women consulting a CAM practitioner consulted with only one or, less frequently, two practitioner types during pregnancy.

**Table 2:** **Different conventional and CAM practitioner professional groups consulted by women for pregnancy**-**related health conditions**

**Practitioners**	Conventional medicine^*^ (**n**=**1366**)	**Complementary medicine**^†^ (**n**=**1629**)
	**%**	**%**
0	0.2	54
1	9.4	25.7
2	48.2	13.1
3	42.2	4.8
4	-	1.8
5	-	0.5
6	-	0.1

^*^ Conventional medicine practitioners includes obstetricians, midwives and general practitioners.

^†^ Complementary medicine practitioners includes acupuncturists, aromatherapists, chiropractors, naturopaths/herbalists, doulas, massage therapists, meditation/yoga classes, and osteopaths.

The women consulted a wide range of health care professionals for a variety of conditions and/or symptoms (see Table 3). The most prevalent condition reported was back pain (39.5%), for which the women most commonly consulted with chiropractors (11.3%) followed by obstetricians (5.9%) and GPs (4.3%). Meanwhile, those women reporting tiredness (35.4%) predominantly consulted with their obstetrician (6.2%) and GP (4.7%). Other than for back pain (4.1%), women mostly consulted with acupuncturists to help prepare for labour (2.4%) and with naturopaths for nausea (1.6%). In contrast, massage therapists were rarely consulted for back pain (0.5%) but were seen for sciatica (6.6%), neck pain (5.9%) and hip pain (4.5%).

**Table 3:** **Patterns of consultations with conventional and CAM practitioners for pregnancy**-**related conditions** (**n**=**1835**)

**Condition**		**All women**						
		**Conventional practitioners**			**CAM practitioners**			
		**General practitioner**	**Obstetrician**	**Midwife**	**Chiropractor**	**Acupuncturist**	**Naturopath**	**Massage**
	**%**	**%**	**%**	**%**	**%**	**%**	**%**	**%**
Back pain	39.5	4.3	5.9	4.1	11.3	4.1	1.7	0.5
Tiredness	35.4	4.7	6.2	3.7	0.4	1.0	1.2	1.0
Reflux/Indigestion	34.7	8.6	12.4	5.0	0.2	0.3	1.0	0.1
Nausea	32.9	12.7	10.5	3.9	0.4	1.3	1.6	0.2
Sciatica	22.1	4.6	5.1	2.9	5.3	1.3	0.2	6.6
Preparing for labour	21.9	3.7	11.9	16.2	1.0	2.4	0.9	0.9
Hip pain	20.9	3.8	5.9	4.4	5.0	1.1	0.1	4.5
Leg cramps	18.2	3.2	4.5	2.9	0.3	0.2	0.6	0.9
Constipation	16.7	4.6	4.9	2.9	0.1	0.5	0.5	0.1
Headache	16.0	5.2	3.7	1.9	2.9	0.4	0.2	1.9
Haemorrhoids	15.8	5.7	4.1	2.1	0.0	0.1	0.4	0.0
Sleeping problems	15.2	2.6	2.8	1.7	0.3	0.5	0.7	0.3
Neck pain	12.4	0.7	0.9	0.7	5.7	0.4	0.2	5.9
Repeated Vomiting	11.0	6.7	5.1	2.0	0.2	0.5	0.4	0.1
Vaginal bleeding	10.4	6.2	7.0	1.7	0.1	0.1	0.1	0.0
Varicose veins	9.4	2.9	3.6	2.5	0.2	0.1	0.2	0.1
Fluid retention	8.7	1.8	3.3	2.0	0.1	0.5	0.3	0.4
Anaemia	7.4	3.8	4.5	2.0	0.0	0.0	0.3	0.3
High Blood Pressure	6.6	3.3	5.3	2.3	0.1	0.2	0.0	0.0
Cravings	6.3	0.4	0.2	0.5	0.1	0.0	0.3	0.0
Dizziness or fainting	6.3	2.9	2.5	1.1	0.1	0.1	0.1	0.1
Weight management	5.5	2.0	1.7	0.6	0.1	0.1	0.2	0.0
Gestational diabetes	4.9	1.7	3.7	1.5	0.0	0.1	0.1	0.0
Urinary Tract Infection	4.9	3.7	1.3	0.5	0.0	0.1	0.0	0.0
Pre-eclampsia	3.2	1.3	3.3	1.6	0.1	0.1	0.2	0.0

Table 4 reports the patterns of women with pregnancy-related health condition consulting with any practitioner from a conventional maternity or CAM professional group, or a combination of practitioners from each group. Amongst the women who reported a pregnancy-related health conditions, many only consulted with a CAM practitioner - 74.6% of women with neck pain, 40.4% of women with sciatica and 35.4% of women with hip pain. Those with back pain were more likely to consult with both conventional and CAM practitioners (61.8%). The majority of women did not seek support from any health professionals for common discomforts such as cravings (81.9%) and tiredness (65.6%). Midwives, GPs and obstetricians were consulted without the inclusion of CAM practitioners for vaginal bleeding (95.8%), high blood pressure (93.4%), pre-eclampsia (93.1%), anaemia (84.6%) and urinary tract infections (83.3%). Gestational diabetes was also associated with the use of conventional practitioners (64.4%) but a significant number of women (22.2%) consulted with both conventional and CAM practitioners for this condition. Pre-eclampsia was the only condition for which no women consulted with CAM practitioners in isolation.

**Table 4:** **Patterns of consultations with conventional and CAM practitioners by women with pregnancy**-**related health conditions**

**Condition**	**Only women with pregnancy**-**related condition**			
	Sought no support^*^	**CAM practitioner only**^†^	**Both CAM and conventional practitioners**^ǂ^	**Conventional practitioner only**^§^
	**%**	**%**	**%**	**%**
Back pain (n=725)	3.9	2.2	61.8	32.1
Tiredness (n=649)	65.6	6.0	2.3	26.0
Reflux/Indigestion (n=637)	40.8	6.0	1.9	51.3
Nausea (n=604)	42.2	5.0	4.6	48.2
Sciatica (n=406)	20.7	40.4	16.3	22.7
Preparing for labour (n=401)	3.2	11.0	13.7	72.1
Hip pain (n=304)	21.9	35.4	17.5	25.3
Leg cramps (n=334)	53.3	7.8	2.1	36.8
Constipation (n=307)	41.4	6.5	1.3	50.8
Headache (n=293)	33.1	21.8	7.9	37.2
Haemorrhoids (n=289)	36.7	4.5	1.4	57.4
Sleeping problems (n=279)	57.4	7.9	4.7	30.1
Neck pain (n=228)	14.9	74.6	5.3	5.3
Repeated Vomiting (n=201)	42.2	5.0	4.6	48.2
Vaginal bleeding (n=191)	2.1	0.5	1.6	95.8
Varicose veins (n=172)	28.5	2.3	3.5	65.7
Fluid retention (n=160)	41.9	5.0	5.0	48.1
Anaemia (n=136)	11.0	0.7	3.7	84.6
High Blood Pressure (n=121)	5.8	0.8	0.0	93.4
Cravings (n=116)	81.9	3.5	0.9	13.8
Dizziness or fainting (n=115)	34.8	3.5	1.7	60.0
Weight management (n=102)	36.3	4.9	5.9	52.9
Gestational diabetes (n=90)	6.7	6.7	22.2	64.4
Urinary Tract Infection (n=90)	13.3	1.1	2.2	83.3
Pre-eclampsia (n=58)	1.7	0.0	5.2	93.1

^*^Women who did not report seeking support from a health professional for the designated pregnancy-related condition.

^†^Women who only consulted with a CAM practitioner for the designated pregnancy-related condition. This includes chiropractors, acupuncturists, naturopaths and massage therapists.

^‡^Women who consulted with both a conventional and CAM practitioner for the designated pregnancy-related condition.

^§^ Women who only consulted with a conventional practitioner for the designated pregnancy-related condition.

### Factors associated with women’s use of CAM during pregnancy

Women consulting CAM practitioners had different consultation patterns with specific conventional practitioners (Table 5). Women who consulted an acupuncturist (p<0.001) during pregnancy visited a GP less frequently than women not consulting an acupuncturist. Women using the services of a doula consulted an obstetrician less frequently than those women who did not use a doula (p<0.001). In contrast, women who had more frequent visits with a midwife were more likely to consult an acupuncturist (p<0.001) or doula (p<0.001). Women’s levels of satisfaction with the care provided by their conventional maternity providers had little impact on their consultation patterns with CAM practitioners during pregnancy (data not shown).

**Table 5:** **The relationship between consultations with CAM practitioners and the number of visits with conventional maternity carers** (**n**=**1835**)

**Practitioner**	**Visits with general practitioner**						**Visits with obstetrician**						**Visits with midwife**					
	**None**	**1 or 2**	**3 or 4**	**5**+	**P value**	***χ***^***2***^	**None**	**1 or 2**	**3 or 4**	**5**+	**P value**	***χ***^***2***^	**None**	**1 or 2**	**3 or 4**	**5**+	**P value**	***χ***^***2***^
Acupuncturist																		
No (%)	93.4	91	83.3	93.4	<0.001^a^	20.6	87.3	89.7	92.0	91.4	0.23	4.3	93.3	94.2	89.8	88.8	<0.001^c^	18.9
Yes (%)	6.6	9	16.7	6.6			12.7	10.3	8.0	8.6			6.7	5.8	10.2	13.6		
Aromatherapist																		
No (%)	99.4	99.5	99.4	99.2	0.89	0.6	99.6	99.5	100.0	99.3	0.78	1.1	99.6	99	99.4	99.6	0.66	1.6
Yes (%)	0.6	0.5	0.9	0.8			0.4	0.5	0.0	0.7			0.4	1	0.6	0.4		
Chiropractor																		
No (%)	85.5	84.9	83.1	80.7	0.29	3.8	79.7	81.8	80.2	85.2	0.11	6.0	86.4	80.8	84.5	93.3	0.2	4.6
Yes (%)	14.6	15.2	17	19.3			20.3	18.2	19.8	14.8			13.6	19.2	15.5	16.7		
Naturopath																		
No (%)	96.3	92.4	89.7	94.1	0.05	7.7	92.1	91.2	95.5	93.1	0.51	2.3	93.3	94.1	94.7	90.0	0.08	6.8
Yes (%)	3.7	7.6	10.3	5.9			7.9	8.8	4.6	6.9			6.7	5.9	5.3	10.0		
Doula																		
No (%)	99.4	98.8	97.8	98.7	0.57	2.0	95.8	96.7	100	99.6	<0.001^b^	27.8	99.8	100	97.6	96.6	<0.001^c^	23.2
Yes (%)	0.6	1.2	2.2	1.3			4.2	3.3	0	0.4			0.2	0	2.4	3.4		
Massage therapist																		
No (%)	66.7	65.1	62.6	70.5	0.16	5.1	70.3	66.2	64.4	64.1	0.34	3.3	93.3	94.2	89.8	65.2	0.85	0.8
Yes (%)	33.3	34.9	37.4	29.5			29.8	33.8	35.7	35.9			6.7	5.8	10.2	34.8		
Meditation/yoga																		
No (%)	87.9	85.6	83.3	88.8	0.21	4.5	84.3	85.9	89.2	86.4	0.66	1.6	99.6	99.0	99.4	83.9	0.19	4.8
Yes (%)	12.1	14.4	16.7	11.2			15.7	14.2	10.8	13.6			0.4	1.1	0.6	16.1		
Osteopath																		
No (%)	95.2	93.6	89.8	96.4	0.009	11.6	94.2	96.3	92.0	93.4	0.35	3.3	86.4	80.8	84.5	93.8	0.87	0.7
Yes (%)	4.8	6.4	10.2	3.6			5.8	3.7	8.0	6.6			13.6	19.2	15.5	6.2		

^a^ Statistically significant association with general practitioner consultations for pregnancy-related health conditions (p<0.005).

^b^ Statistically significant association with obstetrician consultation for pregnancy-related health conditions (p<0.005).

^c^ Statistically significant association with midwife consultation for pregnancy-related health conditions (p<0.005).

## Discussion

This study of a large, nationally representative sample of Australian women who had recently given birth provides the first examination of consultancy patterns across conventional maternity care providers and CAM practitioners during pregnancy. The study presents four key findings. First, the study reveals a substantial level of CAM practitioner use with nearly half of the pregnant women consulting a CAM practitioner concurrent to conventional maternity care. This finding highlights the supplementary nature of CAM use during pregnancy, in line with results from previous studies of CAM consumption both specific to women [4] and in the wider population [8,9].

Second, within the wider pattern of concurrent care, we identified a more complex relationship between the two broader provider groups – for high users of GPs, consultation with some CAM practitioners (eg. acupuncturists) is associated with less frequent visits with a GP. It is possible that this finding reflects a change in women’s health-seeking behaviour as a result of what they perceive as a discouraging response by their GPs to their concerns or preferences [26,27]. It may also highlight a discord between what pregnant women seek [5] and what some GPs may consider unhelpful or irrelevant [28,29]. Alternatively, this finding may be due to a perception amongst these pregnant women that GPs are not core to their maternity care needs (instead addressing such needs with CAM practitioner services), although earlier work suggests that such a view is unlikely to be encouraged by the majority of CAM providers [30].

Third, the findings reveal that frequent midwifery care users are more likely to consult acupuncturists and doulas. This finding supports previous research identifying midwives as a popular source of CAM information for pregnant women [5] and often encouraging CAM use for women in their care [31]. Alternatively, this finding could suggest that women choosing different models of maternity care also hold different values and approaches to CAM use, an issue identified in more general CAM utilisation research [2] but still requiring further investigation in relation to maternity care [32]. Previous research identifies midwives as referring to a range of CAM practitioners - naturopaths/herbalists, homeopaths, chiropractors, osteopaths and massage therapists [16]. The difference between these results and our study findings may be due to the various political and cultural contexts affecting CAM (e.g. political legitimacy) and midwifery (e.g. structure of maternity care provision) across different health systems.

Fourth, our analysis of consultation patterns for the management of specific pregnancy-related conditions suggests pregnant women are making discretionary decisions regarding whom to consult depending on their immediate health concerns. Chiropractors are frequently consulted for back pain and sciatica, massage therapists consulted more commonly for neck pain, and naturopaths and acupuncturists more likely to be consulted for pregnancy-related nausea. Women are consulting with CAM practitioners most commonly for management of pain-related conditions. This may be due to women’s perceptions of CAM treatments as safer (while being equally effective) than conventional pain management [5]. However, this perception is only held when the condition is self-assessed by the women as low risk to them or their babies and women are only rarely consulting with CAM practitioners for more serious complications. Attempts to complement conventional treatments with the care of other therapists still occur - we identified a substantial rate of concurrent CAM and conventional practitioner use amongst pregnant women with gestational diabetes - and this may be the result of women seeking an improved prognosis for these serious conditions and/or a more active role in maintaining their health [5].

Our results highlight a substantial level of CAM practitioner use during pregnancy and a pattern of selective use across different CAM practitioner groups for different health conditions. Our study findings illustrate the inconsistent relationship between the available clinical evidence and the CAM practitioners used by pregnant women. Whilst there is partial alignment between some of the CAM practitioners consulted and the limited existing clinical evidence there are also a number of women consulting CAM practitioners for specific conditions despite an absence of clinical evidence. This underlines concerns that women may be accessing unsafe and ineffective practices. In order to help inform safe, effective and coordinated maternity care that reflects the full breadth of practitioner consultations amongst pregnant women, future research must include examination of decision-making and communication between pregnant women and their maternity care providers about CAM practitioner use. The absence of sufficient clinical evidence regarding many commonly used CAM practices during pregnancy also requires urgent attention.

The main strengths of this study are the high response rate, sample size and national representative sample of pregnant women [5]. This is also the first study to provide insights into the relationship between women’s consultation practices with CAM and conventional care providers for pregnancy-related health conditions. The interpretation of our findings is potentially limited by the fact that health care utilisation is self-reported by the participants and as such our results may be open to the effects of recall bias. In addition, the medical conditions and symptoms were defined by self-report and the lack of confirmatory diagnosis could potentially bias findings. Previous research in this area has identified recall bias is more likely to have affected participants self-report of health conditions related to maternal health during pregnancy such as nausea and vaginal bleeding [33] whilst other more general aspects of health and care provision are less affected [34]. Despite this the ALSWH is a respected source of data for epidemiological research relating to women’s health in Australia, and these limitations are far outstripped by the opportunities provided from conducting the first analysis of CAM and conventional practitioner use amongst a large, nationally representative sample of pregnant women.

## Conclusions

The results from our study have implications for patient safety, access and coordination of maternity care. The study identifies possible barriers to the disclosure and regular communication of CAM use to key members of women’s wider maternity care team posing a potential challenge to effective, inter-professional maternity care across the conventional/CAM practitioner divide [35]. There is a pressing need to facilitate open discussion and disclosure regarding CAM practitioner and CAM use between pregnant women and their maternity care providers.

The research on which this paper is based was conducted as part of the Australian Longitudinal Study on Women’s Health. We are grateful to the Australian Government Department of Health and Ageing (DOHA) and the Australian Research Council (DP1094765) for funding and to the women who provided the survey data.

## Competing interests

We declare that no authors have real or potential conflicts of interest related to this study.

## Authors' contributions

All the authors contributed to the drafting and revision of the article for important intellectual content, and approved the final version to be published. AS had full access to all the data in the study and is the study guarantor. JA, DS, AB, and CG were responsible for the study concept and design. AS and DS were responsible for the data analysis. All the authors participated in the interpretation of data.

## Details of ethics approval

This project has obtained ethical approval from the University of Newcastle (#H-2010_0031), University of Queensland (#2010000411) and the University of Technology Sydney (#2011-174N), and all participants gave informed consent before taking part.

## Funding

This research was funded via an Australian Research Council Discovery Project grant (DP1094765).

## Author details

1Faculty of Health, UTS, Level 7, Building 10, 235-253 Jones Street, Ultimo, New South Wales 2006, Australia. 2School of Social Science, University of Queensland, St Lucia, Queensland 4072, Australia.

## Pre-publication history

The pre-publication history for this paper can be accessed here:

http://www.biomedcentral.com/1471-2393/12/146/prepub
